# Nurses' Trials—Temper

**Published:** 1887-03-05

**Authors:** 


### Words of Consolation.

[Communications for this column should be addressed, "Chaplain," care of the Editor of The Hospital, who will gratefully welcome any suggestions or inquiries from matrons, nurses, and the staff generally.]

### XXIII.—Nurses' Trials—Temper.

However highly qualified a woman may be for the office of a nurse in other respects, she makes a grievous mistake in undertaking it unless her temper is at least fairly under control. A year's probation, or less, has probably convinced many a one, who was not conscious before of much failure in this respect, that she has endless temptations to irritability to contend with in hospital life. There are some people born with a temperament naturally placid and equal, not therefore easily disturbed: but only a very few even of these can trust to this natural endowment without the sustaining power of grace, when they come to be really tried. How much more do the rest of us need the preventing power of daily grace to battle with this trial; considering that we find ourselves so easily put out, or growing impatient, or giving unkind hasty replies when overworked. Here then is another argument for a high standard of religion amongst nurses. We have already made some remarks upon this subject for patients to observe. It may often be advisable for nurses to point out to the sick how much their sufferings are enhanced by indulging irritability; but in order to do so effectively nurses must first be "in possession of their own souls." Now, we believe it to be very common for persons living subject to constant annoyances and provocation to brood over and grieve over their losses of temper. They feel distinctly and acutely that they themselves are the losers. They deplore the suffering brought upon themselves by the extra strain which a day, or even an hour, of ill-temper has imposed. They make resolutions not to be beaten by their enemy again. But all this, without sufficiently recognising that the cure must be a work of grace. They do not believe sufficiently that meekness is essentially a temper, a condition of mind, to be provided by the Holy Ghost and sustained by His indwelling. Hence, with all their brave resolutions there is a lack of cooperation with God. A few moments set apart for prayer over this special difficulty will make a great difference if persevered with. You will come forth from the Sanctuary refreshed and supplied with a strength beyond your own. Fear not, ye who suffer in this temptation, if ye have faith. Take refuge on your knees, at the first opportunity, at any symptom of a rising storm, and ye shall hear words of peace spoken by the still, small voice. Or, if ye care not awhile to kneel, offer a short ejaculatory prayer, "Lord heal me!" "Good Lord deliver me!"

Sir Isaac Newton's temper, it is said, was so equal and mild, that no accident could disturb it; a remarkable instance of which is related as follows:—Sir Isaac had a favourite little dog, which he called Diamond. Being one evening called out of his study into the next room, Diamond was left behind. When Sir Isaac returned, having been absent but a few minutes, he had the mortification to find that Diamond had overturned a lighted candle among some papers, the nearly finished labour of many years, which were soon in flames, and almost consumed to ashes. This loss, from Newton's advanced age, was irreparable; but, without at all punishing the dog, he exclaimed, "Oh, Diamond, Diamond! you little know the mischief you have done!"

If God will help a man so to possess his soul for the sake of a dog, how much more will He help you who have to bear with the provocation of human infirmity; how much more will He clothe you with the ornament of a meek and quiet spirit, even though you are provoked every day.

(*To be continued.*)

